# Low Immune Cross-Reactivity between West Nile Virus and a Zika Virus Vaccine Based on Modified Vaccinia Virus Ankara

**DOI:** 10.3390/ph15030354

**Published:** 2022-03-15

**Authors:** Nereida Jiménez de Oya, Patricia Pérez, Ana-Belén Blázquez, Estela Escribano-Romero, Mariano Esteban, Juan-Carlos Saiz, Juan García-Arriaza, Miguel A. Martín-Acebes

**Affiliations:** 1 Department of Biotechnology, Instituto Nacional de Investigación y Tecnología Agraria y Alimentaria (INIA), Consejo Superior de Investigaciones Científicas (CSIC), 28040 Madrid, Spain; jdeoya@inia.es (N.J.d.O.); blazquez@inia.es (A.-B.B.); eescribano@inia.es (E.E.-R.); jcsaiz@inia.es (J.-C.S.); 2 Centro Nacional de Biotecnología (CNB), Consejo Superior de Investigaciones Científicas (CSIC), 28049 Madrid, Spain; pperez@cnb.csic.es (P.P.); mesteban@cnb.csic.es (M.E.); 3 Centro de Investigación Biomédica en Red de Enfermedades Infecciosas (CIBERINFEC), 28029 Madrid, Spain

**Keywords:** West Nile virus, Zika virus, MVA, vaccine, cross-protection, immunogenicity, antibody-dependent enhancement of infection, vaccine safety

## Abstract

Zika virus (ZIKV) is a mosquito-borne flavivirus whose infection in pregnant women is associated with a spectrum of birth defects, which are together referred as Congenital Zika Syndrome. In addition, ZIKV can also induce Guillain–Barré syndrome, which is an autoimmune disease with neurological symptoms. The recent description of the first local infections of ZIKV in the European continent together with the expansion of one of its potential vectors, the Asian tiger mosquito *(Aedes albopictus)*, invite us to be prepared for future outbreaks of ZIKV in this geographical region. However, the antigenic similarities of ZIKV with other flaviviruses can lead to an immune cross-reactivity with other circulating flaviviruses inducing, in some cases, flavivirus-disease exacerbation by antibody-dependent enhancement (ADE) of infection, which is a major concern for ZIKV vaccine development. Until now, West Nile virus (WNV) is the main medically relevant flavivirus circulating in the Mediterranean Basin. Therefore, anticipating the potential scenario of emergency vaccination against ZIKV in areas of Europe where WNV is endemic, in this investigation, we have evaluated the cross-reactivity between WNV and our previously developed ZIKV vaccine candidate based on modified vaccinia virus Ankara (MVA) vector expressing ZIKV structural proteins (MVA-ZIKV). To this end, mice were first immunized with MVA-ZIKV, subsequently challenged with WNV, and then, the ZIKV- and WNV-specific immune responses and protection against WNV were evaluated. Our results indicate low cross-reactivity between the MVA-ZIKV vaccine candidate and WNV and absence of ADE, supporting the safety of this ZIKV vaccine candidate in areas where the circulation of WNV is endemic.

## 1. Introduction

The potential of emerging viral diseases to cause massive outbreaks with devastating effects on human health and the economy has become clear by the SARS-CoV-2 pandemic that has highlighted the need to anticipate viral emergencies and the utility of safe and efficacious vaccines to control emerging viral diseases. However, before the appearance of SARS-CoV-2, other emerging viruses had threatened humankind during the last decades [1]. One of the most significant concerns is Zika virus (ZIKV), which is a mosquito-borne flavivirus that caused a massive viral outbreak only seven years ago [2]. ZIKV infection in pregnant women is associated with a spectrum of birth defects (including microcephaly), which are together referred as Congenital Zika Syndrome. In addition, ZIKV can also induce Guillain–Barré syndrome, which is an autoimmune disease cursing with neurological symptoms. The 2015–2016 ZIKV epidemic in the Americas and the Caribbean clearly demonstrated the potential of this emerging virus to cause explosive outbreaks when it was introduced into novel geographical areas. The epidemic rapidly waned, but there is a high risk of ZIKV emergence and transmission in other parts of the world [3]. In fact, explosive ZIKV outbreaks have been reported in Yap State, Federated States of Micronesia in 2007, or in French Polynesia in 2013 [4]. In addition, recent reports also point to endemic ZIKV transmission in other regions of Asia [5], and even sporadic local transmission in Europe [6].

During the last years, a huge effort has been made to develop ZIKV vaccines (reviewed in [7]). An important aspect that should be considered for flavivirus vaccine design is the close antigenic relationships among different flaviviruses, which in some cases lead to cross-protection against heterologous flavivirus [8]. However, one of the major concerns for the massive usage of ZIKV vaccines is that this possible cross-reactivity can lead, in other cases, to flavivirus-disease exacerbation by antibody-dependent enhancement (ADE) of infection [9,10]. ADE occurs when suboptimal neutralizing or non-neutralizing cross-reactive antibodies elicited in a primary infection with a flavivirus bind heterologous flavivirus particles during a subsequent infection. This facilitates virus entry and infection into Fcγ receptor-bearing cells and promotes disease exacerbation. This phenomenon has been mainly associated to cross-reactive antibodies directed against prM and the fusion loop epitope within the E protein [11,12]. As the occurrence of heterologous cross-protection or disease exacerbation is the product of complex multiple factors, it should be addressed to warrant the safety of flavivirus vaccine candidates [13,14,15].

The current expansion in Europe of *Aedes albopictus*, the Asian tiger mosquito, which is a potential vector for ZIKV, together with the first local cases of ZIKV in the European continent [6] invites us to be prepared for future ZIKV outbreaks in this region. By now, the flavivirus West Nile virus (WNV) is the main medically relevant mosquito-borne viral threat in European and Eastern Mediterranean countries, being responsible for recurrent outbreaks of febrile illness and meningoencephalitis [16,17]. Interestingly, a diverse degree of cross-reactivity between ZIKV and WNV has been reported, going from a lack of cross-reactive immunity to ADE of infection, or even to heterologous cross-protection [8,14,18,19,20,21,22]. Therefore, ZIKV vaccine candidates that could be used to combat hypothetical future ZIKV outbreaks should consider the potential cross-reactions between WNV and ZIKV. 

Thus, anticipating the potential scenario of emergency vaccination against ZIKV in areas where WNV is circulating, such as some parts of Europe and the Mediterranean Basin, we have evaluated the cross-reactivity between our previously developed ZIKV vaccine candidate based on modified vaccinia virus Ankara (MVA) vector expressing ZIKV structural proteins (termed MVA-ZIKV) [23,24] and WNV. Our results indicate low cross-reactivity between MVA-ZIKV and WNV and absence of ADE in mouse models. Although further work is needed to confirm these results in non-human primates, the present data support the safety profile of this ZIKV vaccine candidate in areas where the circulation of WNV is endemic.

## 2. Results and Discussion

### 2.1. Immunization with MVA-ZIKV Vaccine Candidate Does Not Affect WNV Infection

**Figure 1:**
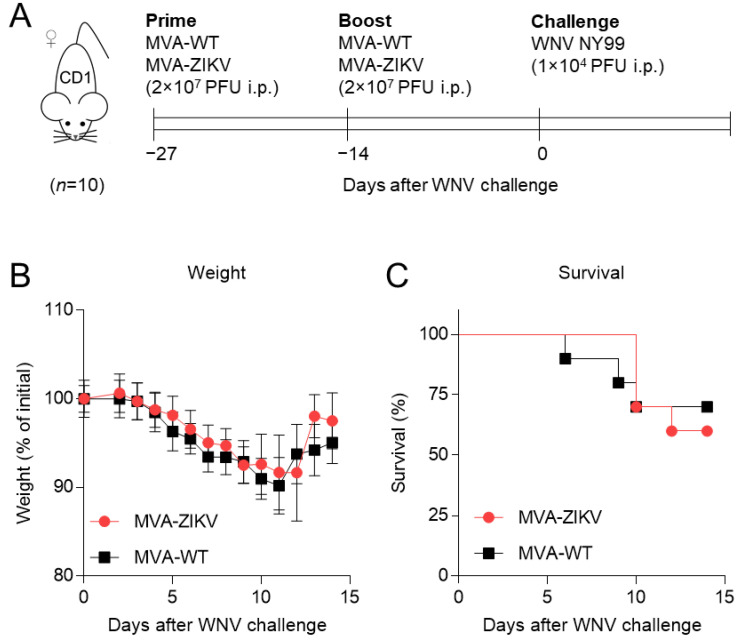
Experimental design, body weight loss, and survival analysis. (**A**) Experimental design. Four-week old CD1 female mice (*n* = 10/group) were vaccinated with two doses of MVA-ZIKV encoding ZIKV prM and E proteins, or with MVA-WT as a control, at days 0 and 13 and challenged with WNV 27 days after the first immunization. Animals were monitored for body weight and survival after WNV challenge, and surviving animals were euthanized 14 days after challenge. (**B**) Body weight loss upon WNV infection. Data are presented as mean ± SEM. (**C**) Kaplan–Meier survival analysis.

We have previously shown that a ZIKV vaccine candidate based on the highly attenuated poxvirus MVA expressing ZIKV prM and E structural proteins (MVA-ZIKV) induced a robust specific protective immune response against ZIKV infection in mouse models [23,24]. Here, we have further evaluated in vivo the consequences of the potential cross-reactivity between vaccination with MVA-ZIKV and the subsequent infection with WNV in a mouse model for WNV infection. To this end, we followed a two-dose immunization schedule similar to that previously performed to test the MVA-ZIKV vaccine efficacy against ZIKV infection in mice [23]. Groups of CD1 female mice (*n* = 10/group) were immunized with two doses of MVA-ZIKV or wild-type MVA vector (MVA-WT, used as control of immunization) at days 0 and 13 and subsequently challenged with WNV at day 27 after the first immunization (14 days post-boost) (Figure 1A). The effect of the MVA-ZIKV vaccine was monitored daily along the time by measuring the body weight and survival of the mice until 14 days post-WNV infection. As shown in Figure 1B, the body weight loss was similar in those mice vaccinated with MVA-ZIKV or with the control MVA-WT vector. Similarly, no statistically significant differences in mice survival upon WNV challenge were noticed regardless of the immunogen administered (Figure 1C). 

These results suggest a low capacity of the MVA-ZIKV vaccine candidate to induce a cross-reactive immune response toward a WNV infection under the conditions assayed. We have previously described that a prior ZIKV infection induces partial cross-protection against a subsequent challenge with WNV in mice [8]. The results reported here could indicate that failure of the MVA-ZIKV vaccine candidate to fully protect against WNV could be due to missing ZIKV non-structural proteins in the vaccine and/or an effect of active ZIKV infection. In fact, there are other studies supporting that non-structural proteins can also contribute to cross-protection against heterologous flaviviruses [18,25]. The different effects of MVA-ZIKV vaccine candidates expressing structural and non-structural proteins have been previously described [23,26,27] and the robust protective effect of immunization with MVA expressing the ZIKV NS1 was demonstrated [27]. In the case of MVA expressing structural glycoproteins, it is noticeable that when compared to other vectored vaccines, some MVA-based ZIKV-vaccines expressing prM and/or E exhibited only a modest level of anti-ZIKV-antibodies [26]. However, the MVA-ZIKV vaccine candidate here analyzed elicited both humoral and potent and polyfunctional ZIKV-specific CD8+ T cell responses [23]. Remarkably, immunization with MVA-ZIKV did not exhibit a negative impact on WNV disease progression in mice, supporting the safety profile of this ZIKV vaccine candidate if used in areas where both viruses co-circulate.

### 2.2. Vaccination with MVA-ZIKV Does Not Alter the WNV-Specific Humoral Response Elicited by WNV Infection

**Figure 2:**
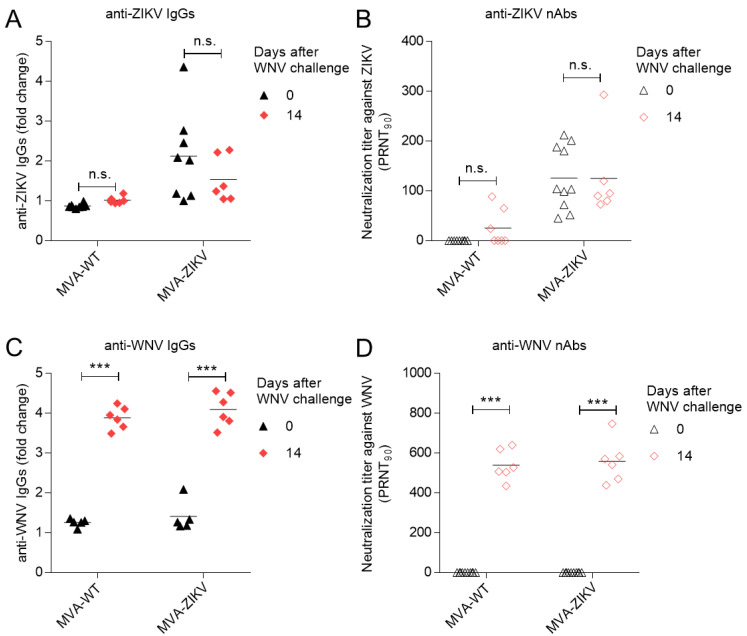
Analysis of antigen-specific humoral responses elicited in CD1 mice vaccinated with MVA-WT or MVA-ZIKV and challenged with WNV. (**A**) Induction of anti-ZIKV IgG antibodies in mice immunized with MVA-ZIKV or MVA-WT and analyzed by ELISA. Values correspond to the fold change over negative serum samples obtained before immunization. (**B**) Induction of anti-ZIKV neutralizing antibodies (nAbs). Data are expressed as the reciprocal serum dilution that neutralized 90% of ZIKV (PRNT_90_). (**C**) Induction of anti-WNV IgG antibodies analyzed by ELISA. (**D**) Induction of anti-WNV nAbs. Each symbol in the graphs denotes a single animal. Three asterisks (***) denote statistically significant differences (*p* < 0.0001) and n.s. indicates not statistically significant differences. Two-way ANOVA Tukey’s multiple comparison was performed with Graphpad Prism 7.0. For experimental design, see panel (A) in Figure 1.

Flaviviruses share antigenic immunodominant epitopes that give rise to cross-reactive antibodies with different affinities in subsequent immunizations or infections with related flaviviruses and/or serotypes [28]. As mentioned above, one detrimental effect that impacts vaccine development is the ADE phenomenon. However, cross-reactive antibodies from B cells primed by one flavivirus can also be protective against another flavivirus if they can efficiently neutralize the virus. To know if there is cross-reactivity of the antibodies elicited by the MVA-ZIKV vaccine candidate against WNV, the ZIKV- and WNV-specific humoral immune responses induced were evaluated by ELISA and virus neutralization in serum from immunized mice (Figure 2).

At 14 days post-WNV challenge, levels of total ZIKV-E-specific binding IgG antibodies induced by MVA-ZIKV vaccination did not significantly differ from those elicited previous to the WNV infection (Figure 2A). The titers of anti-ZIKV neutralizing antibodies were also similar to those observed before WNV infection (Figure 2B), suggesting that the ZIKV-specific humoral responses elicited by the MVA-ZIKV vaccine candidate were not affected by subsequent WNV infection. The analysis of the cross-reactive immune responses against WNV elicited by MVA-ZIKV immunization showed that before WNV challenge (27 days after immunization), ZIKV-specific antibodies induced by MVA-ZIKV did not cross-react with WNV, as similar levels of anti-WNV IgG antibodies were obtained in the MVA-WT control group (Figure 2C). Consistently, in mice vaccinated with MVA-ZIKV, the antibodies raised were not able to neutralize WNV (Figure 2D). The specific humoral response elicited 14 days after WNV infection was also similar in both MVA-ZIKV and MVA-WT immunize mice (Figure 2C,D), which suggests that a previous MVA-ZIKV vaccination did not affect the course of the humoral immune response generated by WNV infection. Similar results have been previously shown in mice infected with ZIKV and subsequently challenged with WNV, in which no cross-reactivity between ZIKV-specific antibodies and WNV was observed [8]. 

It is worth noting that we observed variability in the ZIKV-specific humoral responses elicited in mice immunized with the MVA-ZIKV vaccine candidate, with some mice showing low levels of total ZIKV-E-specific binding IgG antibodies and to a lesser extent neutralizing antibodies. However, all mice developed titers of neutralizing antibodies compatible with the control of ZIKV infection [23]. This variability, and the low antibody levels observed in certain vaccinated mice, rather than be an obstacle to evaluate the occurrence of ADE mimics the scenarios occurring during natural infections and vaccination campaigns, since the ADE phenomenon that occurs between other flaviviruses such as ZIKV and Dengue virus (DENV) depends on a narrow range of antibody levels [10], with higher titers not always being implicated. Whereas the cross-reactivity between ZIKV and DENV has been extensively addressed due to the possibility of dengue severity exacerbation [10], the cross-reactions between ZIKV and WNV have not been systematically addressed, and in some cases, the studies have shown opposite results. For example, the possibility of both ADE or cross-protection in vitro between ZIKV and WNV has been documented [19,21]. In the case of in vivo experiments in the mice model, ZIKV infection exacerbation by pre-existing WNV immunity was proposed [20], although recent reports do not further support this possibility in pregnant mice [22]. Even more, we have previously documented that cross-protection against subsequent WNV challenge by previous ZIKV infection can occur [8]. This variety of results may be explained by multiple factors such as the viral antigens expressed that may differ from those selected for vaccination to the whole panel produced in natural infections, the potency of the immune response, or the immunization schedule. The low cross-reactivity between vaccination with a MVA-ZIKV vaccine candidate only expressing prM and E ZIKV structural proteins and subsequent challenge with WNV described in this study further supports the lack of detrimental effects derived from immune cross-reactivity between WNV and ZIKV found in other studies.

Overall, these results evidence the safety profile of the MVA-ZIKV vaccine candidate against subsequent WNV infection. A lack of cross-reactivity in terms of antibodies generated by the MVA-ZIKV vaccine able to react with WNV antigens, together with the absence of effect on disease progression and survival, support the use of such type of vaccine in regions in which both ZIKV and WNV co-circulate.

## 3. Materials and Methods

### 3.1. Viruses

The origin and procedures for amplification in chicken embryo fibroblasts (CEF) and titration of attenuated MVA-WT and recombinant MVA-ZIKV viruses have been previously described [23,24]. WNV NY99 (GenBank accession KC407666.1) was produced and titrated in Vero CCL81 cells (ATCC) by plaque assay in standard agarose semisolid medium, as previously described [8]. Virus titers were expressed as plaque-forming units (PFU)/mL.

### 3.2. Mice Experiments

Experimental infections were conducted at the Biosafety level-3 animal facilities of the Centro de Investigación en Sanidad Animal (CISA-INIA-CSIC, Valdeolmos, Madrid, Spain). The study was approved by the Ethics Committee of Animal Experimentation of INIA and by the Division of Animal Protection of the Comunidad de Madrid (PROEX 05/14). Animals were handled in strict accordance with the guidelines of the European Community 86/609/CEE. Groups of four-week-old Hsd:ICR CD1 female mice (*n* = 10/group, Envigo) were immunized intraperitoneally (i.p.) with two doses (2 × 10^7^ PFU/animal) of MVA-ZIKV or MVA-WT (used as control of immunization) at days 0 and 13. On day 27 after the first immunization (14 days post-boost), mice were challenged i.p. with 1 × 10^4^ PFU/animal of WNV NY99. Animals were kept with ad libitum access to food and water and monitored daily for weight loss and clinical signs of WNV infection. Animals exhibiting clear signs of WNV infection were anesthetized under isoflurane and humanely euthanized. All surviving animals were anesthetized and humanely euthanized at 14 days post-challenge.

### 3.3. Antibody Analyses

ZIKV-E-specific binding IgGs were determined by ELISA using a recombinant ZIKV E protein (Fitzgerald Industries International) as a coating antigen. Anti-WNV IgG antibodies were determined using an in-house developed ELISA based on heat-inactivated WNV-infected Vero cell lysates [8]. ZIKV and WNV-neutralizing antibody titers were determined by a standard plaque reduction neutralization test, as previously described [8], and are expressed as the reciprocal serum dilution that neutralized 90% of ZIKV (PRNT_90_).

## Data Availability

Data is contained within the article.
